# Tissue Engineering and Regenerative Medicine: Semantic Considerations for an Evolving Paradigm

**DOI:** 10.3389/fbioe.2014.00057

**Published:** 2015-01-12

**Authors:** Ravi Katari, Andrea Peloso, Giuseppe Orlando

**Affiliations:** ^1^Wake Forest School of Medicine, Winston-Salem, NC, USA; ^2^General Surgery, Fondazione IRCCS Policlinico San Matteo Pavia, University of Pavia, Pavia, Italy

**Keywords:** tissue engineering, regenerative medicine, stem cells, organ bioengineering, device regulation, commercialization

## Abstract

Tissue engineering (TE) and regenerative medicine (RM) are rapidly evolving fields that are often obscured by a dense cloud of hype and commercialization potential. We find, in the literature and general commentary, that several of the associated terms are casually referenced in varying contexts that ultimately result in the blurring of the distinguishing boundaries which define them. “TE” and “RM” are often used interchangeably, though some experts vehemently argue that they, in fact, represent different conceptual entities. Nevertheless, contemporary scientists have a general idea of the experiments and milestones that can be classified within either or both categories. Given the groundbreaking achievements reported within the past decade and consequent watershed potential of this field, we feel that it would be useful to properly contextualize these terms semantically and historically. In this concept paper, we explore the various definitions proposed in the literature and emphasize that ambiguous terminology can lead to misplaced apprehension. We assert that the central motifs of both concepts have existed within the surgical sciences long before their appearance as terms in the scientific literature.

## Introduction

We are fascinated by a closing sentence in Umberto Eco’s novel “The Name of the Rose”: *Stat rosa pristina nomine*; *nomina nuda tenemus* (Umberto Eco, 2012). Roughly translated, “the primordial rose endures only in its name; and empty names are all we have.” When we apply this idea of “empty names” to the terms tissue engineering (TE) and regenerative medicine (RM), we realize these are actually concepts that escape us. A related term, namely, stem cell – is similarly afflicted with the stigma of “empty names” due to the dramatic differences between the categories which fall under its umbrella, i.e., the embryonic stem cell (ESC) versus the adult stem cell (ASC). Certainly, we know the experiments and innovations upon which to apply the terms, but discerning the common, fundamental elements which describe them is a complicated endeavor. Even more challenging is discerning the crucial attributes which differentiate the terms that are often used interchangeably in the literature.

In a previous concept paper (Orlando et al., 2011a), we have endorsed the following definition: “RM is the field in health sciences that aims to replace or regenerate human cells, tissues, or organs in order to restore or establish normal function. The process of regenerating body parts can occur *in vivo* or *ex vivo* and may require cells, natural or artificial scaffolding materials, growth factors, gene manipulation, or combinations of all the above mentioned elements.” Furthermore, “TE is a subfield of RM, which is narrower in scope and strictly defined as the manufacturing of body parts *ex vivo*, by seeding sells on and/or into a supporting scaffold. All bioengineered organs that have been implanted in human beings so far have been manufactured using TE technologies.” We offered definitions in order to consolidate a scattered terminology for our own ease of understanding and organization. In other words, these interpretations, though viable for our own ends and purposes, were not grounded in hard empiricism and/or semantics.

Indeed, our claim that TE is a subfield of RM is partially contradicted by the fact that the regular appearance of the former term in the literature preceded that of the latter by almost 15 years (Wolter and Meyer, 1984). Still, some experts opine that it is the unique combination of technological approaches associated with RM that distinguishes it from TE. Daar and Greenwood, for example, assert that “although TE is an important component of RM according to our definition, we believe that RM as a whole is not the same as TE and is not exclusively dedicated to using its methods. For instance, while TE commonly uses a combination of cells, biomaterials, and soluble molecules to encourage cell and tissue growth, RM includes this but may also involve simply the genetic engineering of cells followed by their transplantation without the use of biomaterial scaffolds, or even the pharmaceutical targeting of developmental pathways of stem cells as a means of therapy (Daar and Greenwood, 2007).” Furthermore, the authors outline criteria with which they are able to reject the definitions proposed by other experts. For example, definitions that focus too much on cells or natural components are excluded because RM can include non-cell based therapies such as synthetic materials releasing soluble molecules.

Other definitions and characterizations are readily available in the literature, or are endorsed by governmental institutions for regulatory purposes. The National Institute for Biomedical Imaging and Bioengineering^1^ claims that TE “refers to the practice of combining scaffolds, cells, and biologically active molecules into functional tissues,” with the goal being to “assemble functional constructs that restore, maintain, or improve damaged tissues or whole organs.” On the other hand, the Institute defines RM as “a broad field that includes tissue engineering but also incorporates research on self-healing – where the body uses its own systems, sometimes with help [from] foreign biological material to recreate cells and rebuild tissues and organs.” Interestingly, they conclude that the two terms have become interchangeable “as the field hopes to focus on cures instead of treatments for complex, chronic diseases.” Correspondingly, some authors find no need to distinguish the two terms, RM and TE, and lump them together unambiguously. An editorial in PNAS written by Badylak and Nerem (2010), for example, discusses “progress in TE and RM” and treats them as references to the same category of research endeavors. Similarly, Fisher and Mauck (2013) recently penned a review paper which put forward utilized a combination acronym to simplify the reference to the “broad discipline [singular] of tissue engineering and regenerative medicine (TERM).” Nevertheless, the review ultimately suggests that TE emphasizes the starting materials and scaffolds used to create *de novo* tissue implants whereas RM emphasizes endogenous tissue formation that may occur secondary to induction from the starting materials.

Naturally, the distinctions begin to appear rather arbitrary. Consequently, even the definition we put forward no longer seems satisfactory. Why should some technologies be included while others excluded? Perhaps the more important question is, why is a proper definition needed at this time? In this opinion paper, we will address these pertinent issues in a series of didactics.

## Problem of Names: TE and RM

As evidenced by our definition above, there has been much debate and comment regarding the difference between RM and TE. Sometimes they are used interchangeably and sometimes definitions given for one are reminiscent of definitions given for the other which fuels the growing confusion. The Committee on the Biological and Biomedical Applications of Stem Cell Research has stated that “regenerative medicine seeks to understand how and why stem cells, whether derived from human embryos or adult tissues, are able to develop into specialized tissues, and seeks to harness this potential for tissue-replacement therapies that will restore lost function in damaged organs (Commission on Life Sciences, 2002).” Elsewhere, TE is described similarly as “the persuasion of the body to heal itself, through the delivery to the appropriate sites, of molecular signals, cells, and supporting structures (Williams, 1999).” Here, we have come to the first of several conflicts which stem from the *existence of names* and the walls which separate them.

**Figure 1:**
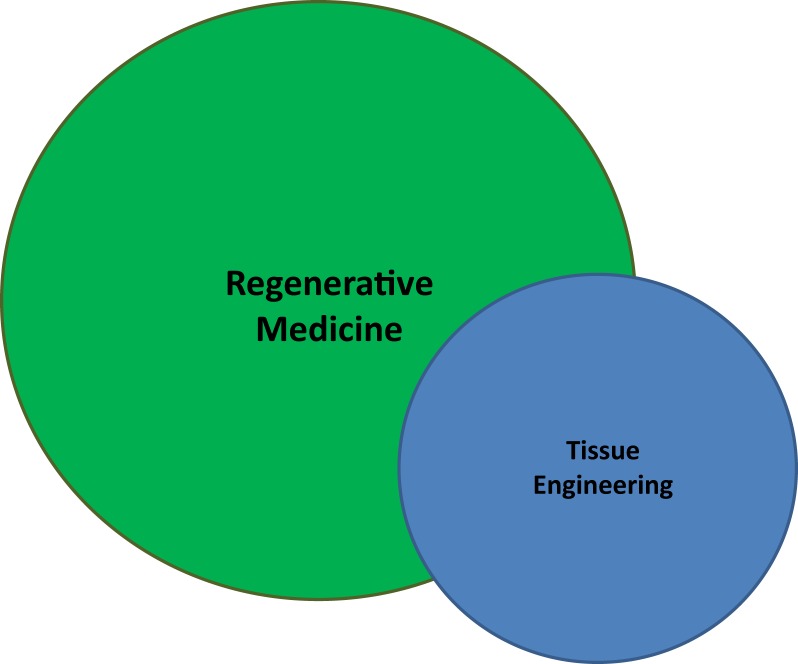
**A diaphragmatic illustration of the relationship between regenerative medicine (RM) and tissue engineering (TE)**. Though RM is a broader and more generalized field than TE, one does not wholly encompass the other. Both seek to restore function, but TE is more narrow in its focus and does not require cellular regeneration. Nevertheless, taken together, RM/TE has grown to resemble a singular research entity.

Both terms refer to a body of literature and scientific investigation seeking to replace or restore physiological function by encouraging the body to return to equilibrium, hence *regenerative* medicine. As a functional convention, we can exploit the “engineering” component of term TE to rationally differentiate it by underscoring the controlled introduction, growth, and/or manufacture of functional material which would restore a lost or damaged deficit, both anatomically and functionally. By this construction, perhaps TE and RM simply represent two different ways of interpreting or evaluating a common entity (Figure 1).

Still, the subtle distinctions within TE and RM in other contexts are worth examining. Suppose, we were to surgically implant a renal scaffold obtained from the decellularization of a discarded human kidney, which our group has successfully performed and characterized (Orlando et al., 2013). Upon treatment with the appropriate induction factors and successful reattachment to the existing vascular and urinary system, the construct begins to spontaneously develop into a viable, cellularized, kidney, which is physiologically assimilated by the recipient. Such a development in the field has not yet occurred, but it is conceivable given previous successes in the implantation of acellular matrices (Chen et al., 1999; Murphy and Atala, 2012). Given the straightforward use of proliferating cells to regenerate a kidney, this project would meet the criteria for virtually all definitions of RM, but perhaps not for TE. Some of this process occurred *ex vivo*, namely, the controlled decellularization; however, the second component of this processed occurred *in vivo*, namely, the regeneration itself. Though it would meet the criteria for Williams’ definition of TE (Williams, 1999), it is not clear as to whether it does so for the criteria that our group previously put forward, i.e., that the manufacture takes place outside of the body. The most important observation to take away from this thought experiment is how arbitrary these categories are. If, instead, the acellular construct was allowed to mature in a bioreactor that facilitated cell seeding, attachment, and proliferation *ex vivo* (i.e., complete regeneration) and then reconnected to the patient’s circulatory and urinary systems, does the difference merit an entirely new term? Would it not suffice to simply classify both as just RM and just TE?

We find that the trivial concerns borne by this conflict far outweigh the convenience that distinguishing the terms offers. Even if there were wide agreement on separate definitions for the two concepts, it is likely to be of little value. It is acceptable for RM and TE not to be synonymous, with the former emphasizing the cellular regenerative aspect of tissue replacement and the latter emphasizing the engineering and manufacturing aspects of tissue replacement (Salgado et al., 2013). Technological advances in the last decade make clear that these two categories, though different, are extremely related. Correspondingly, we are already witnessing a sensible, organic tendency to combine the terms and treat them as a single research pursuit (e.g., “TERM”) (Salgado et al., 2013).

## Influence of Stem Cells in RM/TE

Fortunately, the literature demonstrates that, in addition to defining terms, there have been attempts to justify the need for such acute terminology. These justifications mostly underscore the legal, ethical, and regulatory frameworks through which RM/TE research will have to navigate in order to successfully blossom and commercialize (Kellathur and Lou, 2012). At the moment, the hype is growing due to an industry that has exploded in scale and commercial productivity in the last decade (Lysaght and Hazlehurst, 2004; Lysaght et al., 2008). We find, in current discourse, a sense of urgency behind establishing an appropriate regulatory framework within which the transition of RM/TE innovations from bench to bedside can occur (Mcallister et al., 2012; Bailey et al., 2014). Indeed, much of the difficulty in doing so can be attributed to the controversy associated with the use of biologically active cells and their introduction into human patients (Ancans, 2012). Yet, the evidence for the inherent novelty of modern regenerative therapies is unconvincing.

That cell therapy has been a core historical element in RM/TE is well understood. Up till the mid-1970s, human biology was cell-focused, but the paradigm changed completely when it became clear that the extracellular matrix is as important as the cells (Slavkin and Greulich, 1975). That idea was exemplified in the famous Harvard mouse (Vacanti et al., 1988), which represented the conceptual paradigm of an apparently new field that was coined “TE” (Wolter and Meyer, 1984). The Harvard mouse paved the ground for seminal research that ultimately led to clinical translation via the transplantation of bioengineered vessels, segments of the urinary tract, and upper airways, bones, cartilage, and skin. We have seen that in the last two decades, the discovery of stem cells has revolutionized this field of health sciences which evolved into modern RM.

What is it about RM/TE that separates it from organ transplantation, a skin graft, or a blood transfusion? Indeed, that a blood transfusion is a simple, but definitive example of RM can hardly be contested. Functional cells from another human being are introduced into a deficient recipient to serve as temporary replacement; regenerated, autologous cells eventually take their place. The difference, however, lies in its frequent use of stem cells (versus somatic cells) as the both the fuel and driver of tissue regeneration. And here, we come to another manifestation of the *problem of names*. Stem cells immediately bring to mind a catalog of tensions which heavily inform public and popular discourse. Understanding the term properly, however, is necessary to deflate the hype and controversy that arise when people think of RM, which inevitably brings to mind the employment of stem cells. What are controversial are ESCs. ASCs are not embryonic, are not found in the same location, and exhibit a different array of functions and capabilities. It is an unfortunate happenstance that they share a nominal stem, as it were.

Another useful thought experiment would be to consider a state of affairs in which these two had two totally discrete names: much in the fashion of, to invoke one example, a neuron and a hepatocyte. ASCs serve a natural regenerative function *in situ*, aside from their use and manipulation in the laboratory; ESCs do not. ASCs are constantly expanding, differentiating, and sloughing *in vivo* throughout the human life whereas ESCs represent a single, highly transient stage of development. They share a capacity for differentiation, though the systems in which they exert this function differ drastically. ASCs are regenerative by nature, i.e., in most cases, they exist to regenerate: those which reside in the hair follicle, the dermis, and the bone marrow are prime examples. ESCs, however, are developmental by nature and there is no temporal overlap whatsoever between their existence and the physiological functioning of organ systems. That the two cell types differ primarily by their position on a spectrum of differentiation is an interpretational construction. It is reasonable to regard them as two totally categorically different cell types even though they are similarly applied in RM/TE investigations. In so far as stem cells serve as the chief element of controversy within RM/TE, the dominant contribution of ESCs and not ASCs to these circumstances needs to be taken into account.

## Classifying RM/TE: Lessons from Transplant Surgery

Indeed, the hype surrounding RM/TE strategies needs to be recontextualized, from a regulatory standpoint. There has been much concern in the regards to the lack of bureaucratic oversight over their introduction into clinical practice (European Commission, 2001). As we have just discussed, the principal apprehensions include the ethical and legal implications of using biologically active cells of potentially non-self origin and/or with the ability to renew and multiply uncontrollably (e.g., stem cells). Upon consideration of current surgical practice, however, it is clear that the methods and goals of RM/TE have been roughly approximated and conceptually applied for many generations, which precede the appearance of the terms within the literature. We argue that organ transplantation and reconstructive surgery are forms of RM/TE in that their manipulation and reintroduction of functional, living tissues from non-self donors (Orlando et al., 2013b). Though surgical transplantation is far older than RM/TE as a conceptual entity, it still represents an iteration of RM/TE principles as they are currently understood. Thus, the assertion that RM/TE is a recent development is contradicted by the history of transplant and reconstructive surgery.

**Figure 2:**
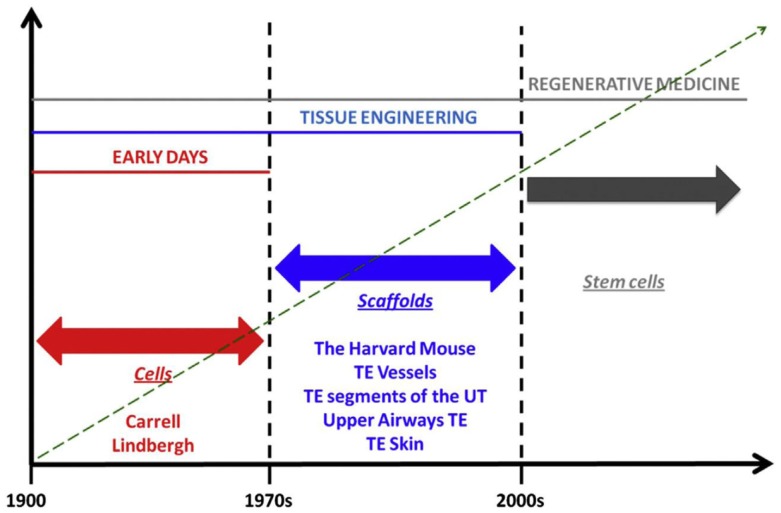
**Phases in the history of regenerative medicine**. When observing the evolution of regenerative medicine era, three phases can be identified. The first phase spans from the early days to the 1970s. In those days, Alexis Carrel and Charles Lindbergh for the first time had the idea of growing organ outside the human body. For those visionary experiments, Carrel should be referred as father, pioneer, and precursor of concepts that are currently being developed in modern regenerative medicine. In those days, biology was “cytocentric” and cells were considered to be the only relevant players in the biology of complex viable systems. Things changed when it was understood that actually the extracellular matrix is as important as cells, in organ welfare; this intuition allowed transition to the second phase which spans from the 1970s to the discovery of stem cells. This intuition was conceptualized by the iconic Harvard mouse, which represents the paradigm of new ideas that paved the ground for a breakthrough in the history of medicine, namely, the bioengineering and implantation of relatively simple body parts like vessels, segments of the urinary tract, and upper airways, bones, skin, and cornea. The third phase began with the discovery of stem cells, wherein the term regenerative medicine has been coined. The discovery of stem cells made us realize that, despite complex organisms like mammals have lost during phylogenesis their ability to regenerate in full their body parts, yet, these cells – if manipulated appropriately – may re-confer us this quiescent ability. In fact, the ultimate goal of regenerative medicine is to max out the regenerative, reparative potential intrinsic to the human body [adapted from Katari et al. (2014), with permission].

Alexis Carrel is considered the father of transplant surgery, but his seminal work on cell culture and *ex vivo* organ preservation and growth anticipated organ bioengineering and regeneration concepts that would not be fully realized for decades (Figure 2). The perfusion pump that he and Charles Lindbergh, celebrated aviator and engineer, developed allowed organs to exist outside of the body during surgery; it symbolizes a crucial step in the developmental timeline of the modern bioreactor, an important component of several RM/TE technologies. Furthermore, the commonalities are abundant and obvious. Skin grafts and engineered skin substitutes are variations of the same technology. Both seek to restore function by capitalizing on the body’s ability to regenerate itself; furthermore, the former introduces living cells and – in the case of full-thickness grafts – even ASCs. Organ transplantation involves procurement and implantation of foreign tissues, which are eventually assimilated into the recipient’s physiology. Furthermore, these grafts, broadly speaking, are far from perfect. Extended criteria donors and donations after cardiac death form a crucial buffer in the chronic shortage of transplantable organs. However, these issues are largely immunized from the scrutiny that RM/TE technologies are subjected to; indeed, they are considered to fall within categorically separate disciplines.

In this context, we have previously argued for a recontextualization of organ transplantation and TE – taken together – as a synergistic paradigm that has always existed (Orlando et al., 2013b,c). For our assertions regarding TE, we employed the interpretation that includes the manipulation of mammalian somatic cells and biomaterial scaffolds while excluding auxiliary methods such as gene therapy and pharmacology. This categorical distinction cannot be emphasized enough. The European Union, for example, has attempted to initiate and guide the fruition of RM/TE commercialization and trade via an umbrella classification, Advanced Therapy Medicinal Products: a regulatory construct that includes gene therapy, somatic cell therapy, and tissue-engineered products (European Medicines Agency, 0000). Such regulatory efforts have been criticized as cumbersome and clumsy (Pearce et al., 2013). In the United States, the Food and Drug Administration’s (FDA) Center for Biologics Evaluation and Research has endeavored similarly by exercising extreme caution in the evaluation of investigational new drug applications: “Early experiences with cellular and gene therapy (CGT) products indicate that some CGT products may pose substantial risks to subjects.^2^” Notable here is the grouping together of gene and cell therapies within the same risk category. Given the clear overlap between TE methods and transplant surgery, which is regulated by the Health Resources Services Administration (Lee et al., 2010), a more realistic interpretation that takes its cues from the history of transplantation could enhance commercialization pipelines and research efforts while assuaging common safety concerns.

## Conclusion

To be sure, the manufacturing challenges posed by cell based regeneration and biomaterials are correctly warrant stringent GMP regulations, particularly if a primary aim is to expand undifferentiated human cells on a massive scale. Nevertheless, the introduction of non-self progenitor cells is not a radical approach; in fact, it occurs in hundreds of thousands of patients annually as is immediately manifest to any transplant practitioner. Bone marrow transplants, blood transfusions, skin grafts, and even renal transplantation (Oliver et al., 2004; Bussolati et al., 2005) all introduce allogeneic cells with regenerative capacities into the recipient. Furthermore, the regenerative cells in these grafts *restore the regenerative function* which constituted their original purpose in the donor’s physiology. For example, the dermal stem cell niche that is transferred into the recipient within a full-thickness graft continues to carry out its renewal responsibilities post-operatively and facilitates graft survival (Alonso and Fuchs, 2003; Fuchs, 2007). The trajectory of hematopoietic stem cells in transplanted marrow is similarly exemplifying. Yet, these therapies were never formally considered to exist within the domain of RM/TE.

If the criteria for categorical distinction involve the alteration of the harvested tissue pre-operatively, then we should recall the numerous examples of pre-infusion and pre-graft modifications that occur routinely in clinical practice. Citrate is added as a chelating agent to packed red blood cells to prevent coagulation. An even more dramatic example is the skin graft mesher and tissue expander routinely used by plastic surgeons to promote vascularization and cell growth in order to achieve better outcomes in larger wound beds (Colman and Gurucharri, 1987). If the term “biomaterial” refers to the introduction of synthetic material to aid the regenerative process by providing a biomolecular and spatial environment conducive to cell proliferation and vascularization, then could not the ubiquitous Band-Aid, the common suture, or the vascular stent be considered elements of RM/TE? Indeed, much like these examples, modern RM/TE solutions have incorporated biodegradable scaffolds designed to withdraw as the formation of new tissue takes place (Matsumura et al., 2003). The point is that RM/TE has been employed by surgical operators long before it became a discrete field in need of novel regulatory oversight and commercialization strategies.

We wholeheartedly agree with the claim by Hollander et al. (2009) that, without a doubt, “never has there been a more exciting time to be involved in surgical science.” Recent success with the tissue-engineered airway is illustrative (Gonfiotti et al., 2014). The premise, however, has been understood for more than a century, as we attempted to make clear here. It is simply the methods of acquiring therapeutic tissues that has undergone rapid evolution in recent decades – fueled largely by a growing understanding of undifferentiated precursor cells. Furthermore, stem cell transfer has been taking place long before our newfound understanding of their nature and regional niches. It is against this background that we argue that a recontextualization of RM/TE research is warranted. It should be obvious that the restoration and replacement of diseased tissue via the introduction of healthier grafts has existed long before the terms we now use to describe it. It is our feeling that contemporary discourse would benefit from the incorporation of this truism.

## Conflict of Interest Statement

The authors declare that the research was conducted in the absence of any commercial or financial relationships that could be construed as a potential conflict of interest.

## Footnotes

^1^http://www.nibib.nih.gov/science-education/science-topics/tissue-engineering-and-regenerative-medicine

^2^http://www.raps.org/focus-online/news/news-article-view/article/3734
